# Supplementary material for: Genome-Wide Analysis of Specific PfR2R3-MYB Genes Related to Paulownia Witches’ Broom
Source: Genes (Basel). 2022 Dec 20;14(1):7. doi: 10.3390/genes14010007 (PMC9858720; doi:10.3390/genes14010007)
Supplement: Supplementary file 1 [file genes-14-00007-s001.zip › Supplementary Materials Tables S1.pdf]

Table S1. The primers used for qRT-PCR in PF and PFI

Gene	Forward primer sequences (5'-3')	Reverse primer sequence (5'-3')
PfR2R3-MYB3	AGCCCTCCTTTTCACAGGTC	TGGCCCTATTGTTGGCTACTG
PfR2R3-MYB5	GGCACAATGGGAGAGTGCTA	CCTGCCCGGATGTATTCAGT
PfR2R3-MYB15	TGTCCTGATAATATGTTGT	AGCAGAAGGAAGTAATAC
PfR2R3-MYB18	CGGATCTCAAGCGAGGCAAC	GCACCTTTCTCTAATCGGGCT
PfR2R3-MYB24	CACAACGGCCATGACAACTG	ACCAGAATCATCCCCCGAGA
PfR2R3-MYB49	AGCCTGACATTAAGCGTGGAA	TTGGATACTGGTGAAGGGGC
PfR2R3-MYB116	CGCCTGAAGAAGAGCAAACC	ATTGGGGTCCTGATTCCCAC
PfR2R3-MYB128	CCGCTGGAACTCTCTTGCTC	AGGTGGTAAGTAGTGGGGGT
PtActin	GTATCCACGAGACTACTTACAA	CCACCACTGAGCACAATA
PfRAX2	ACCACCAACAACTTCATA	GACAACATATTATCAGGACAA

PF, *Paulownia fortunei* seedlings; PFI, phytoplasma-infected *Paulownia fortunei* seedlings.
