# Supplementary material for: Genome-Wide Analysis of Specific PfR2R3-MYB Genes Related to Paulownia Witches’ Broom
Source: Genes (Basel). 2022 Dec 20;14(1):7. doi: 10.3390/genes14010007 (PMC9858720; doi:10.3390/genes14010007)
Supplement: Supplementary file 1 [file genes-14-00007-s001.zip › Supplementary Materials Tables S2.pdf]

Table S2 Primers of 35S::*PfR2R3-MYB15-GFP* and 35S::*GFP* were designed

Gene	Forward primer (5′-3′)	Reverse primer (5′-3′)
PfR2R3-MYB15-GFP	GTGGATCCAAAGAATTCATGGGAAGAGCACCTTGCTG	CTCCTTTACCCATGAATTCTGGATAGGCAAAATTGAG
GFP	TTCCAAGTTACCATCCATT	TCCAAGCATATATTCCCATT
