# Supplementary material for: Genome-Wide Analysis of Specific PfR2R3-MYB Genes Related to Paulownia Witches’ Broom
Source: Genes (Basel). 2022 Dec 20;14(1):7. doi: 10.3390/genes14010007 (PMC9858720; doi:10.3390/genes14010007)
Supplement: Supplementary file 1 [file genes-14-00007-s001.zip › Supplementary Materials Tables S5.pdf]

Table S6 Identification of *R2R3-MYB* gene family members of paulownia and analysis of their physicochemical properties.

Gene name	Gene ID	CDS/bp	Strand	Amino acid	Relative molecular mass	Atomic composition	Isoelectric point	Subcellular location
PfR2R3-MYB1	Paulownia_LG1G000802	1065	−	354	40,194.04	$C_{1759}H_{2743}N_{491}O_{557}S_{16}$	5.52	Nucleus
PfR2R3-MYB2	Paulownia_LG1G000867	741	+	321	36,000.41	$C_{1576}H_{2448}N_{444}O_{492}S_{16}$	6.36	Nucleus
PfR2R3-MYB3	Paulownia_LG1G000885	1134	−	377	42,538.42	$C_{1840}H_{2869}N_{565}O_{570}S_{16}$	9.04	Nucleus
PfR2R3-MYB4	Paulownia_LG0G000043	939	+	312	35,605.89	$C_{1540}H_{2414}N_{472}O_{474}S_{15}$	8.71	Nucleus
PfR2R3-MYB5	Paulownia_LG0G000230	954	−	317	35,588.85	$C_{1551}H_{2423}N_{439}O_{493}S_{15}$	6.35	Nucleus
PfR2R3-MYB6	Paulownia_LG0G000419	954	−	317	35,426.97	$C_{1588}H_{2465}N_{427}O_{481}S_{6}$	6.16	Nucleus
PfR2R3-MYB7	Paulownia_LG0G000672	1050	+	349	38,818.62	$C_{1687}H_{2641}N_{479}O_{534}S_{20}$	5.37	Nucleus
PfR2R3-MYB8	Paulownia_LG0G000840	1632	+	543	59,030.37	$C_{2563}H_{3939}N_{705}O_{844}S_{28}$	4.81	Nucleus
PfR2R3-MYB9	Paulownia_LG0G001590	837	−	278	30,326.13	$C_{1309}H_{2124}N_{394}O_{418}S_{8}$	7.74	Nucleus
PfR2R3-MYB10	Paulownia_LG0G001904	759	−	252	28,170.72	$C_{1212}H_{1945}N_{367}O_{383}S_{12}$	8.77	Nucleus
PfR2R3-MYB11	Paulownia_LG2G000207	1143	+	380	42,945.20	$C_{1872}H_{2907}N_{565}O_{565}S_{18}$	8.98	Nucleus
PfR2R3-MYB12	Paulownia_LG2G000208	843	+	280	32,093.00	$C_{1404}H_{2171}N_{425}O_{419}S_{12}$	9.26	Nucleus
PfR2R3-MYB13	Paulownia_LG2G000288	1056	+	351	39,717.55	$C_{1739}H_{2699}N_{483}O_{548}S_{18}$	5.62	Nucleus
PfR2R3-MYB14	Paulownia_LG2G000521	666	−	221	25,659.39	$C_{1146}H_{1810}N_{318}O_{335}S_{8}$	8.99	Nucleus
PfR2R3-MYB15	Paulownia_LG2G000709	843	+	280	30,922.59	$C_{1360}H_{2104}N_{368}O_{433}S_{12}$	5.45	Nucleus
PfR2R3-MYB16	Paulownia_LG2G001188	1017	+	338	37,868.27	$C_{1653}H_{2542}N_{468}O_{520}S_{18}$	7.89	Nucleus
PfR2R3-MYB17	Paulownia_LG2G001328	1083	−	360	41,173.18	$C_{1818}H_{2922}N_{534}O_{533}S_{12}$	9.06	Nucleus
PfR2R3-MYB18	Paulownia_LG3G000069	771	+	256	28,659.42	$C_{1233}H_{1989}N_{375}O_{386}S_{13}$	9.09	Nucleus
PfR2R3-MYB19	Paulownia_LG3G000553	1143	−	380	42,535.77	$C_{1871}H_{2912}N_{542}O_{570}S_{13}$	9.39	Nucleus
PfR2R3-MYB20	Paulownia_LG3G000878	1641	+	521	56,620.01	$C_{2476}H_{3819}N_{679}O_{799}S_{23}$	5.08	Nucleus
PfR2R3-MYB21	Paulownia_LG3G001030	1017	−	338	37,578.49	$C_{1627}H_{2556}N_{464}O_{510}S_{25}$	5.45	Nucleus
PfR2R3-MYB22	Paulownia_LG3G001206	714	+	237	26,901.16	$C_{1201}H_{1849}N_{335}O_{362}S_{4}$	5.73	Nucleus
PfR2R3-MYB23	Paulownia_LG3G001325	1014	+	337	37,409.06	$C_{1623}H_{2561}N_{471}O_{512}S_{17}$	8.27	Nucleus
PfR2R3-MYB24	Paulownia_LG4G000458	756	+	251	28,373.29	$C_{1233}H_{1882}N_{352}O_{399}S_{11}$	4.98	Nucleus
PfR2R3-MYB25	Paulownia_LG4G000782	1653	−	550	59,982.77	$C_{2595}H_{4064}N_{724}O_{857}S_{27}$	5.23	Nucleus
PfR2R3-MYB26	Paulownia_LG4G001152	738	+	245	28,438.88	$C_{1248}H_{1917}N_{355}O_{383}S_{13}$	7.74	Nucleus
PfR2R3-MYB27	Paulownia_LG4G001329	1005	−	334	37,665.39	$C_{1661}H_{2545}N_{451}O_{512}S_{20}$	6.53	Nucleus
PfR2R3-MYB28	Paulownia_LG5G000219	1128	+	375	41,801.67	$C_{1875}H_{2902}N_{504}O_{549}S_{16}$	6.56	Nucleus
PfR2R3-MYB29	Paulownia_LG5G000372	951	−	316	35,503.58	$C_{1547}H_{2412}N_{420}O_{508}S_{15}$	4.75	Nucleus
PfR2R3-MYB30	Paulownia_LG5G000622	837	+	278	31,088.95	$C_{1352}H_{2094}N_{400}O_{412}S_{17}$	9.14	Nucleus
PfR2R3-MYB31	Paulownia_LG5G000817	1140	−	379	43,464.00	$C_{1936}H_{3078}N_{544}O_{561}S_{16}$	9.40	Nucleus
PfR2R3-MYB32	Paulownia_LG5G000892	723	+	240	27,059.57	$C_{1176}H_{1891}N_{343}O_{371}S_{9}$	9.03	Nucleus
PfR2R3-MYB33	Paulownia_LG5G000913	867	−	288	33,025.32	$C_{1458}H_{2304}N_{422}O_{441}S_{7}$	9.15	Nucleus
PfR2R3-MYB34	Paulownia_LG5G000915	894	−	297	34,103.25	$C_{1497}H_{2339}N_{439}O_{458}S_{9}$	8.78	Nucleus
PfR2R3-MYB35	Paulownia_LG5G000961	1050	+	349	39,304.30	$C_{1731}H_{2680}N_{480}O_{532}S_{18}$	6.51	Nucleus
PfR2R3-MYB36	Paulownia_LG5G001256	720	−	239	27,564.86	$C_{1221}H_{1858}N_{352}O_{363}S_{9}$	8.68	Nucleus
PfR2R3-MYB37	Paulownia_LG5G001353	1161	−	386	41,930.41	$C_{1814}H_{2824}N_{530}O_{589}S_{14}$	6.28	Nucleus

Gene name	Gene ID	CDS/bp	Strand	Amino acid	relative molecular mass	Atomic composition	Isoelectric point	Subcellular location
PfR2R3-MYB38	Paulownia_LG5G001363	1104	+	367	41,573.58	$C_{1791}H_{2797}N_{541}O_{558}S_{23}$	8.65	Nucleus
PfR2R3-MYB39	Paulownia_LG5G001379	981	−	326	36,613.66	$C_{1600}H_{2475}N_{447}O_{516}S_{12}$	5.88	Nucleus
PfR2R3-MYB40	Paulownia_LG5G001432	1086	+	361	40,771.64	$C_{1794}H_{2766}N_{496}O_{561}S_{16}$	5.70	Nucleus
PfR2R3-MYB41	Paulownia_LG5G001535	582	−	193	22,101.82	$C_{960}H_{1495}N_{285}O_{295}S_{11}$	7.01	Nucleus
PfR2R3-MYB42	Paulownia_LG5G001541	1173	+	390	44,426.62	$C_{1931}H_{3007}N_{557}O_{612}S_{19}$	5.93	Nucleus
PfR2R3-MYB43	Paulownia_LG5G001628	843	−	280	32,375.35	$C_{1396}H_{2197}N_{409}O_{443}S_{18}$	6.02	Nucleus
PfR2R3-MYB44	Paulownia_LG7G000084	693	+	230	26,488.34	$C_{1155}H_{1818}N_{330}O_{345}S_{20}$	8.13	Nucleus
PfR2R3-MYB45	Paulownia_LG7G000249	1461	−	486	54,619.30	$C_{2352}H_{3758}N_{688}O_{761}S_{24}$	6.39	Nucleus
PfR2R3-MYB46	Paulownia_LG7G000282	1260	−	419	45,929.97	$C_{2002}H_{3061}N_{571}O_{635}S_{20}$	6.08	Nucleus
PfR2R3-MYB47	Paulownia_LG7G000292	1026	+	341	38,132.24	$C_{1677}H_{2651}N_{487}O_{505}S_{13}$	9.24	Nucleus
PfR2R3-MYB48	Paulownia_LG7G000293	1185	+	394	43,748.86	$C_{1945}H_{3037}N_{539}O_{575}S_{18}$	8.13	Nucleus
PfR2R3-MYB49	Paulownia_LG7G000468	873	+	290	33,010.18	$C_{1450}H_{2259}N_{407}O_{448}S_{14}$	7.60	Nucleus
PfR2R3-MYB50	Paulownia_LG7G000770	906	+	301	33,562.14	$C_{1479}H_{2364}N_{422}O_{451}S_{9}$	8.99	Nucleus
PfR2R3-MYB51	Paulownia_LG7G000814	957	−	318	35,611.06	$C_{1582}H_{2469}N_{437}O_{486}S_{7}$	5.99	Nucleus
PfR2R3-MYB52	Paulownia_LG7G001038	822	+	273	31,006.69	$C_{1341}H_{2088}N_{388}O_{426}S_{17}$	5.08	Nucleus
PfR2R3-MYB53	Paulownia_LG7G001184	996	−	331	37,655.15	$C_{1647}H_{2543}N_{467}O_{514}S_{17}$	5.82	Nucleus
PfR2R3-MYB54	Paulownia_LG6G000128	762	+	253	29,095.48	$C_{1257}H_{1969}N_{395}O_{385}S_{10}$	9.11	Nucleus
PfR2R3-MYB55	Paulownia_LG6G000225	1116	−	371	42,415.46	$C_{1852}H_{2860}N_{526}O_{580}S_{20}$	6.10	Nucleus
PfR2R3-MYB56	Paulownia_LG6G000269	1377	−	458	52,527.89	$C_{2341}H_{3559}N_{631}O_{713}S_{18}$	5.28	Nucleus
PfR2R3-MYB57	Paulownia_LG6G000458	978	+	325	36,063.13	$C_{1569}H_{2453}N_{445}O_{508}S_{12}$	5.85	Nucleus
PfR2R3-MYB58	Paulownia_LG6G001343	1176	−	391	43,213.98	$C_{1907}H_{2951}N_{519}O_{581}S_{24}$	5.68	Nucleus
PfR2R3-MYB59	Paulownia_LG6G001386	585	−	194	21,859.59	$C_{949}H_{1517}N_{287}O_{295}S_{6}$	9.28	Nucleus
PfR2R3-MYB60	Paulownia_LG8G000013	999	−	332	37,056.18	$C_{1597}H_{2477}N_{473}O_{511}S_{18}$	6.48	Nucleus
PfR2R3-MYB61	Paulownia_LG8G000599	1155	+	384	43,621.61	$C_{1897}H_{2963}N_{541}O_{610}S_{16}$	5.64	Nucleus
PfR2R3-MYB62	Paulownia_LG8G000927	1056	−	351	39,382.66	$C_{1711}H_{2661}N_{487}O_{557}S_{13}$	5.68	Nucleus
PfR2R3-MYB63	Paulownia_LG8G000997	789	−	262	29,488.34	$C_{1293}H_{2021}N_{377}O_{389}S_{13}$	8.53	Nucleus
PfR2R3-MYB64	Paulownia_LG8G001506	813	−	270	30,945.32	$C_{1375}H_{2073}N_{375}O_{427}S_{8}$	6.07	Nucleus
PfR2R3-MYB65	Paulownia_LG8G001508	906	−	301	34,551.55	$C_{1553}H_{2347}N_{419}O_{469}S_{5}$	6.81	Nucleus
PfR2R3-MYB66	Paulownia_LG8G001510	795	−	264	30,370.20	$C_{1354}H_{2080}N_{372}O_{407}S_{9}$	6.40	Nucleus
PfR2R3-MYB67	Paulownia_LG8G001516	915	+	304	34,856.86	$C_{1561}H_{2362}N_{418}O_{478}S_{7}$	5.75	Nucleus
PfR2R3-MYB68	Paulownia_LG8G001572	1359	+	452	48,990.49	$C_{2147}H_{3304}N_{610}O_{676}S_{16}$	5.57	Nucleus
PfR2R3-MYB69	Paulownia_LG9G000403	951	−	316	35,339.52	$C_{1521}H_{2408}N_{448}O_{491}S_{16}$	7.00	Nucleus
PfR2R3-MYB70	Paulownia_LG9G000404	855	−	284	32,542.59	$C_{1437}H_{2202}N_{406}O_{433}S_{14}$	6.87	Nucleus
PfR2R3-MYB71	Paulownia_LG9G000406	870	−	289	32,829.71	$C_{1440}H_{2219}N_{401}O_{452}S_{14}$	5.70	Nucleus
PfR2R3-MYB72	Paulownia_LG9G000855	1452	+	483	52,860.94	$C_{2281}H_{3671}N_{665}O_{722}S_{28}$	6.62	Nucleus
PfR2R3-MYB73	Paulownia_LG9G000973	2778	−	925	104,983.70	$C_{4550}H_{7375}N_{1323}O_{1453}S_{35}$	5.47	Nucleus
PfR2R3-MYB74	Paulownia_LG10G000032	930	+	309	34,984.53	$C_{1522}H_{2318}N_{450}O_{482}S_{11}$	6.07	Nucleus
PfR2R3-MYB75	Paulownia_LG10G000082	1089	+	362	40,516.23	$C_{1741}H_{2745}N_{519}O_{560}S_{19}$	6.34	Nucleus
PfR2R3-MYB76	Paulownia_LG10G000355	813	+	270	31,025.82	$C_{1353}H_{2093}N_{381}O_{424}S_{17}$	5.69	Nucleus
PfR2R3-MYB77	Paulownia_LG10G000388	858	+	285	32,373.88	$C_{1427}H_{2182}N_{404}O_{447}S_{7}$	6.26	Nucleus

PfR2R3-MYB78	Paulownia_LG10G000389	858	+	285	23,382.04	$C_{1430}H_{2194}N_{408}O_{441}S_{7}$	8.19	Nucleus

Gene name	Gene ID	CDS/bp	Strand	Amino acid	relative molecular mass	Atomic composition	Isoelectric point	Subcellular location
PfR2R3-MYB79	Paulownia_LG10G000430	741	+	246	29,067.75	$C_{1277}H_{1989}N_{387}O_{376}S_{9}$	8.90	Nucleus
PfR2R3-MYB80	Paulownia_LG10G001062	1026	+	341	37,298.08	$C_{1635}H_{2572}N_{476}O_{499}S_{13}$	8.56	Nucleus
PfR2R3-MYB81	Paulownia_LG10G001105	714	+	237	26,334.15	$C_{1123}H_{1771}N_{343}O_{369}S_{11}$	7.64	Nucleus
PfR2R3-MYB82	Paulownia_LG10G001365	924	−	307	34,085.28	$C_{1514}H_{2309}N_{407}O_{464}S_{14}$	5.69	Nucleus
PfR2R3-MYB83	Paulownia_LG10G001370	714	+	237	27,426.81	$C_{1200}H_{1880}N_{370}O_{357}S_{7}$	9.58	Nucleus
PfR2R3-MYB84	Paulownia_LG10G001524	891	−	296	33,075.27	$C_{1438}H_{2258}N_{422}O_{444}S_{16}$	8.84	Nucleus
PfR2R3-MYB85	Paulownia_LG12G000038	972	+	323	35,634.70	$C_{1517}H_{2421}N_{459}O_{500}S_{17}$	5.34	Nucleus
PfR2R3-MYB86	Paulownia_LG12G000233	747	+	248	28,179.51	$C_{1229}H_{1888}N_{348}O_{385}S_{15}$	5.45	Nucleus
PfR2R3-MYB87	Paulownia_LG12G000384	1062	+	353	39,303.93	$C_{1693}H_{2652}N_{486}O_{549}S_{22}$	5.18	Nucleus
PfR2R3-MYB88	Paulownia_LG12G000483	1653	−	550	60,154.67	$C_{2606}H_{4028}N_{734}O_{853}S_{27}$	5.20	Nucleus
PfR2R3-MYB89	Paulownia_LG12G000671	1005	+	334	37,658.00	$C_{1650}H_{2516}N_{454}O_{521}S_{19}$	6.32	Nucleus
PfR2R3-MYB90	Paulownia_LG12G000796	744	+	247	28,711.23	$C_{1259}H_{1941}N_{361}O_{385}S_{13}$	8.32	Nucleus
PfR2R3-MYB91	Paulownia_LG11G000146	1008	+	335	37,465.55	$C_{1655}H_{2585}N_{463}O_{497}S_{17}$	6.63	Nucleus
PfR2R3-MYB92	Paulownia_LG11G000237	909	+	302	34,215.57	$C_{1500}H_{2335}N_{399}O_{478}S_{19}$	4.77	Nucleus
PfR2R3-MYB93	Paulownia_LG11G000431	1017	+	338	37,245.88	$C_{1606}H_{2508}N_{480}O_{498}S_{23}$	9.12	Nucleus
PfR2R3-MYB94	Paulownia_LG11G000525	627	+	204	23,139.23	$C_{1010}H_{1607}N_{291}O_{312}S_{10}$	8.57	Nucleus
PfR2R3-MYB95	Paulownia_LG11G000585	1122	−	373	42,382.33	$C_{1872}H_{2966}N_{532}O_{559}S_{16}$	8.89	Nucleus
PfR2R3-MYB96	Paulownia_LG11G000721	1065	+	354	40,040.94	$C_{1764}H_{2728}N_{502}O_{539}S_{14}$	8.45	Nucleus
PfR2R3-MYB97	Paulownia_LG11G000791	1014	−	337	36,985.03	$C_{1611}H_{2561}N_{467}O_{494}S_{19}$	8.92	Nucleus
PfR2R3-MYB98	Paulownia_LG11G000921	723	−	240	27,760.94	$C_{1218}H_{1870}N_{346}O_{380}S_{10}$	6.91	Nucleus
PfR2R3-MYB99	Paulownia_LG11G000981	1137	−	378	41,022.65	$C_{1780}H_{2783}N_{519}O_{570}S_{14}$	6.38	Nucleus
PfR2R3-MYB100	Paulownia_LG11G000996	978	−	325	36,277.45	$C_{1569}H_{2451}N_{441}O_{513}S_{18}$	5.94	Nucleus
PfR2R3-MYB101	Paulownia_LG11G001072	591	−	196	22,480.13	$C_{973}H_{1509}N_{293}O_{301}S_{11}$	7.74	Nucleus
PfR2R3-MYB102	Paulownia_LG13G000074	591	+	196	22,252.21	$C_{975}H_{1561}N_{285}O_{299}S_{6}$	9.13	Nucleus
PfR2R3-MYB103	Paulownia_LG13G000111	1149	+	382	41,985.07	$C_{1855}H_{2858}N_{510}O_{575}S_{15}$	6.05	Nucleus
PfR2R3-MYB104	Paulownia_LG13G000788	972	−	323	36,038.29	$C_{1571}H_{2454}N_{446}O_{500}S_{14}$	6.02	Nucleus
PfR2R3-MYB105	Paulownia_LG13G000910	1317	+	438	49,657.50	$C_{2179}H_{3358}N_{608}O_{684}S_{20}$	5.36	Nucleus
PfR2R3-MYB106	Paulownia_LG13G000946	741	+	246	28,030.36	$C_{1226}H_{1909}N_{351}O_{384}S_{10}$	7.66	Nucleus
PfR2R3-MYB107	Paulownia_LG13G001006	771	−	256	29,980.44	$C_{1305}H_{2029}N_{407}O_{394}S_{8}$	8.55	Nucleus
PfR2R3-MYB108	Paulownia_LG15G000372	774	−	257	29,419.04	$C_{1281}H_{2026}N_{390}O_{392}S_{8}$	9.19	Nucleus
PfR2R3-MYB109	Paulownia_LG15G000984	1032	+	343	38,276.32	$C_{1683}H_{2655}N_{477}O_{516}S_{14}$	7.63	Nucleus
PfR2R3-MYB110	Paulownia_LG15G001029	1308	+	435	48,880.01	$C_{2133}H_{3290}N_{606}O_{690}S_{13}$	5.47	Nucleus
PfR2R3-MYB111	Paulownia_LG15G001074	1149	−	382	42,475.72	$C_{1889}H_{2957}N_{527}O_{547}S_{21}$	8.66	Nucleus
PfR2R3-MYB112	Paulownia_LG15G001153	705	−	234	26,208.30	$C_{1128}H_{1785}N_{339}O_{360}S_{11}$	9.04	Nucleus
PfR2R3-MYB113	Paulownia_LG16G000157	798	+	265	30,111.35	$C_{1330}H_{2030}N_{380}O_{413}S_{5}$	6.71	Nucleus
PfR2R3-MYB114	Paulownia_LG16G000162	1032	−	343	38,686.47	$C_{1696}H_{2661}N_{481}O_{530}S_{13}$	6.25	Nucleus
PfR2R3-MYB115	Paulownia_LG16G000742	3516	−	1171	129,740.84	$C_{5648}H_{8940}N_{1584}O_{1845}S_{37}$	5.90	Nucleus
PfR2R3-MYB116	Paulownia_LG16G000801	1047	−	348	38,850.15	$C_{1696}H_{2641}N_{477}O_{549}S_{11}$	5.66	Nucleus
PfR2R3-MYB117	Paulownia_LG16G000857	966	−	321	35,652.19	$C_{1562}H_{2456}N_{450}O_{481}S_{13}$	6.67	Nucleus

PfR2R3-MYB118	Paulownia_LG16G001109	1062	−	353	40,023.86	$C_{1752}H_{2737}N_{499}O_{549}S_{14}$	6.31	Nucleus

Gene name	Gene ID	CDS/bp	Strand	Amino acid	relative molecular mass	Atomic composition	Isoelectric point	Subcellular location
PfR2R3-MYB119	Paulownia_LG14G000136	894	−	297	33,915.97	$C_{1487}H_{2318}N_{422}O_{466}S_{11}$	6.10	Nucleus
PfR2R3-MYB120	Paulownia_LG14G000379	1017	−	338	37,750.88	$C_{1652}H_{2612}N_{484}O_{497}S_{17}$	8.97	Nucleus
PfR2R3-MYB121	Paulownia_LG14G000391	1218	+	405	44,932.06	$C_{1960}H_{2970}N_{556}O_{611}S_{26}$	5.71	Nucleus
PfR2R3-MYB122	Paulownia_LG14G000438	1443	+	480	53,944.28	$C_{2334}H_{3688}N_{666}O_{760}S_{22}$	5.59	Nucleus
PfR2R3-MYB123	Paulownia_LG14G000712	684	−	227	26,205.90	$C_{1144}H_{1788}N_{326}O_{343}S_{19}$	6.23	Nucleus
PfR2R3-MYB124	Paulownia_LG18G000728	936	+	311	34,954.82	$C_{1515}H_{2340}N_{434}O_{488}S_{16}$	5.22	Nucleus
PfR2R3-MYB125	Paulownia_LG18G000924	819	−	272	31,066.74	$C_{1353}H_{2084}N_{388}O_{423}S_{16}$	5.25	Nucleus
PfR2R3-MYB126	Paulownia_LG18G001019	621	−	206	23,703.70	$C_{1032}H_{1647}N_{313}O_{317}S_{6}$	9.37	Nucleus
PfR2R3-MYB127	Paulownia_LG18G001150	1176	+	391	43,768.10	$C_{1902}H_{3013}N_{555}O_{602}S_{15}$	7.62	Nucleus
PfR2R3-MYB128	Paulownia_LG17G000049	930	+	309	34,888.50	$C_{1515}H_{2318}N_{448}O_{481}S_{12}$	6.45	Nucleus
PfR2R3-MYB129	Paulownia_LG17G000125	1068	+	355	39,993.62	$C_{1717}H_{2711}N_{507}O_{558}S_{19}$	5.82	Nucleus
PfR2R3-MYB130	Paulownia_LG17G000576	768	+	255	29,393.99	$C_{1277}H_{1991}N_{361}O_{405}S_{16}$	5.68	Nucleus
PfR2R3-MYB131	Paulownia_LG17G000624	834	−	277	31,203.81	$C_{1374}H_{2095}N_{379}O_{429}S_{13}$	5.79	Nucleus
PfR2R3-MYB132	Paulownia_LG17G000682	717	+	238	28,057.58	$C_{1244}H_{1922}N_{372}O_{361}S_{6}$	9.47	Nucleus
PfR2R3-MYB133	Paulownia_LG19G000304	651	+	216	24,446.22	$C_{1071}H_{1657}N_{317}O_{330}S_{6}$	7.03	Nucleus
PfR2R3-MYB134	Paulownia_LG19G000353	2922	+	973	109,162.82	$C_{4722}H_{7660}N_{1370}O_{1536}S_{30}$	5.08	Nucleus
PfR2R3-MYB135	Paulownia_LG19G000502	1383	−	460	50,242.42	$C_{2169}H_{3454}N_{632}O_{697}S_{22}$	6.10	Nucleus
PfR2R3-MYB136	Paulownia_LG19G000573	789	+	262	28,543.75	$C_{1214}H_{1956}N_{382}O_{397}S_{9}$	5.94	Nucleus
PfR2R3-MYB137	Paulownia_LG19G000796	828	+	275	31,260.10	$C_{1374}H_{2123}N_{389}O_{422}S_{13}$	6.59	Nucleus
PfR2R3-MYB138	Paulownia_LG19G000797	963	+	320	35,770.87	$C_{1536}H_{2449}N_{455}O_{504}S_{13}$	6.42	Nucleus
