# Supplementary material for: Genome-Wide Analysis of Specific PfR2R3-MYB Genes Related to Paulownia Witches’ Broom
Source: Genes (Basel). 2022 Dec 20;14(1):7. doi: 10.3390/genes14010007 (PMC9858720; doi:10.3390/genes14010007)
Supplement: Supplementary file 1 [file genes-14-00007-s001.zip › Supplementary Materials Tables S6.pdf]

Table S7 The KA/KS analysis of PfR2R3-MYBs genes.

Seq_1	Seq_2	Ka	Ks	Ka/Ks	Date (MYA)
Pfo11g003610.1	Pfo12g002450.1	0.363444061	5.094258321	0.071343862	275.0899493
Pfo02g019480.1	Pfo04g000700.1	0.03347633	0.388692668	0.086125449	20.98940407
Pfo02g002310.1	Pfo10g004020.1	0.357676046	4.022210846	0.088925235	217.1993857
Pfo05g004610.1	Pfo11g003610.1	0.339917597	3.666148942	0.092717891	197.9720429
Pfo07g008020.1	Pfo18g011550.1	0.054804179	0.561703905	0.097567738	30.33201087
Pfo04g010480.1	Pfo12g003940.1	0.389896807	3.931310261	0.099177318	212.2907541
Pfo05g000500.1	Pfo19g000480.1	0.271511682	2.420721462	0.112161472	130.7189589
Pfo11g000320.1	Pfo19g000480.1	0.045294068	0.392971576	0.115260418	21.2204651
Pfo05g004610.1	Pfo19g005800.1	0.359874222	3.061624075	0.117543569	165.3277001
Pfo04g013370.1	Pfo20g008050.1	0.411131028	3.486890304	0.117907646	188.2920764
Pfo11g004370.1	Pfo19g006890.1	0.076321218	0.626396129	0.121841778	33.82539097
Pfo13g007390.1	Pfo15g010010.1	0.408536082	3.337226678	0.12241784	180.2102406
Pfo07g010710.1	Pfo18g009290.1	0.057162739	0.462263561	0.123658328	24.96223229
Pfo11g010810.1	Pfo15g010910.1	0.092019229	0.693703903	0.132649143	37.46001076
Pfo12g002450.1	Pfo19g005800.1	0.371996629	2.766796439	0.134450306	149.4070077
Pfo06g002230.1	Pfo13g001480.1	0.081738857	0.559937174	0.145978623	30.2366074
Pfo01g008330.1	Pfo03g003220.1	0.070003083	0.45140118	0.155079529	24.37566372
Pfo06g013990.1	Pfo08g004250.1	0.329346964	2.008994762	0.163936198	108.4857171
Pfo02g006850.1	Pfo04g010480.1	0.081999668	0.497890449	0.164694197	26.88608425
Pfo06g008290.1	Pfo13g006030.1	0.093764278	0.56548517	0.16581209	30.53619918
Pfo06g009750.1	Pfo15g010010.1	0.441948189	2.665164547	0.165823979	143.9188855
Pfo13g008090.1	Pfo15g010910.1	0.361788428	2.145989576	0.168588157	115.8834371
Pfo06g013730.1	Pfo13g010060.1	0.067407006	0.390305684	0.172703111	21.07650694
Pfo13g010210.1	Pfo14g008500.1	0.374557481	2.124910008	0.176269809	114.7451404
Pfo11g011230.1	Pfo15g011750.1	0.088846565	0.498925253	0.178075903	26.94196366
Pfo09g006030.1	Pfo16g001650.1	0.345819672	1.889998477	0.182973519	102.0599178
Pfo09g006030.1	Pfo16g011210.1	0.081883828	0.423289301	0.193446486	22.85762225
Pfo06g016400.1	Pfo17g007100.1	0.350996543	1.811696137	0.19373919	97.8315914
Pfo07g012180.1	Pfo18g007310.1	0.085401353	0.433264177	0.197111502	23.39626556
Pfo08g004250.1	Pfo14g008500.1	0.072799872	0.363699186	0.200165067	19.63975604
Pfo11g010810.1	Pfo13g008090.1	0.365078865	1.7837483	0.204669496	96.3224082
Pfo02g016300.1	Pfo15g010910.1	0.487766154	2.294266509	0.21260222	123.8903915
Pfo02g004240.1	Pfo04g012220.1	0.083392545	0.388876399	0.214444861	20.99932555
Pfo07g000840.1	Pfo17g007100.1	0.082508735	0.377975995	0.21829094	20.41070373
Pfo02g002310.1	Pfo20g008050.1	0.374425888	1.705271206	0.2195697	92.08464512
Pfo07g002780.1	Pfo17g004370.1	0.068537292	0.303692657	0.225679781	16.39940348
Pfo03g012350.1	Pfo05g013420.1	0.363993209	1.608005751	0.226363126	86.83231055
Pfo05g013420.1	Pfo12g006860.1	0.0634585	0.279722246	0.226862541	15.10500128
Pfo06g015500.1	Pfo13g010980.1	0.068106922	0.298042271	0.228514304	16.09428263
Pfo06g013990.1	Pfo13g010210.1	0.075487489	0.328647813	0.229691133	17.7469819

Pfo08g001950.1	Pfo14g010110.1	0.06682958	0.290607053	0.229965443	15.69278086
Pfo02g006850.1	Pfo12g003940.1	0.395703826	1.71045464	0.231344238	92.36455056
Pfo11g000840.1	Pfo12g000400.1	0.561675654	2.400839327	0.233949706	129.6453237
Pfo06g014510.1	Pfo08g003540.1	0.555043	2.349245363	0.23626438	126.8592496
Pfo03g012350.1	Pfo12g006860.1	0.397729469	1.625387094	0.244698306	87.77090308
Pfo10g004020.1	Pfo20g008050.1	0.075314061	0.301780437	0.249565749	16.2961436
Pfo11g013900.1	Pfo15g003730.1	0.083176935	0.332010966	0.250524661	17.92859216
Pfo06g013830.1	Pfo17g003900.1	0.588806571	2.316514836	0.254177768	125.0918011
Pfo08g004250.1	Pfo13g010210.1	0.368061189	1.420135117	0.259173359	76.68729632
Pfo06g013830.1	Pfo07g003110.1	0.618933926	2.348795135	0.263511243	126.8349373
Pfo16g001650.1	Pfo16g011210.1	0.402640635	1.502267408	0.268021946	81.12244003
Pfo11g003610.1	Pfo19g005800.1	0.115088131	0.416221075	0.276507216	22.47593805
Pfo09g015360.1	Pfo16g001600.1	0.142374863	0.50227164	0.28346188	27.12266856
Pfo01g008980.1	Pfo03g002780.1	0.141520732	0.491926393	0.287686804	26.56402522
Pfo08g002380.1	Pfo14g009740.1	0.130498455	0.449826149	0.290108646	24.29061205
Pfo07g003110.1	Pfo17g003900.1	0.098668854	0.338309095	0.291652976	18.26869113
Pfo07g005000.1	Pfo17g001350.1	0.125627672	0.428956423	0.292868147	23.16364684
Pfo07g008480.1	Pfo18g012080.1	0.16090086	0.546997261	0.294152954	29.53785209
Pfo07g003210.1	Pfo17g003780.1	0.123154859	0.41301448	0.29818533	22.30278192
Pfo02g011810.1	Pfo04g005500.1	0.172639824	0.565757056	0.305148336	30.55088102
Pfo09g010090.1	Pfo16g008690.1	0.135128219	0.437007174	0.309212817	23.5983874
Pfo09g009390.1	Pfo16g008130.1	0.125134833	0.401813539	0.311425127	21.69793111
Pfo05g007870.1	Pfo12g004970.1	0.108564247	0.340259905	0.31906271	18.37403487
Pfo06g009750.1	Pfo13g007390.1	0.138836968	0.429764683	0.32305346	23.20729288
Pfo12g000400.1	Pfo19g001230.1	0.529426295	1.633407702	0.32412379	88.20401591
Pfo02g002310.1	Pfo04g013370.1	0.10991815	0.327717126	0.335405572	17.6967248
Pfo11g003950.1	Pfo19g006280.1	0.260602706	0.748137129	0.34833548	40.39940497
Pfo08g000980.1	Pfo14g010660.1	0.181763275	0.505251633	0.35974802	27.28358818
Pfo05g004610.1	Pfo12g002450.1	0.133505835	0.361629763	0.369178228	19.5280072
Pfo06g012720.1	Pfo13g009450.1	0.150953364	0.394221071	0.382915514	21.28793783
Pfo01g009160.1	Pfo03g002570.1	0.088381288	0.223244844	0.395893972	12.05522158
Pfo11g000840.1	Pfo19g001230.1	0.135488564	0.34104939	0.397269627	18.41666706
Pfo08g014110.1	Pfo14g000740.1	0.097381314	0.234606907	0.415082894	12.66877298
Pfo10g008590.1	Pfo20g005080.1	0.152434458	0.367188653	0.415139348	19.82818726
Pfo02g008560.1	Pfo04g008950.1	0.122920651	0.267578622	0.459381435	14.44924559
Pfo10g004030.1	Pfo20g008040.1	0.180902165	0.385487018	0.46928212	20.81629897
Pfo06g009270.1	Pfo13g006990.1	0.313236525	0.606206742	0.516715673	32.73516407
Pfo08g013680.1	Pfo14g001120.1	0.154180683	0.282637794	0.54550625	15.26244088
