# Supplementary figures and images for: Genome-Wide Analysis of Specific PfR2R3-MYB Genes Related to Paulownia Witches’ Broom
Source: Genes (Basel). 2022 Dec 20;14(1):7. doi: 10.3390/genes14010007 (PMC9858720; doi:10.3390/genes14010007)

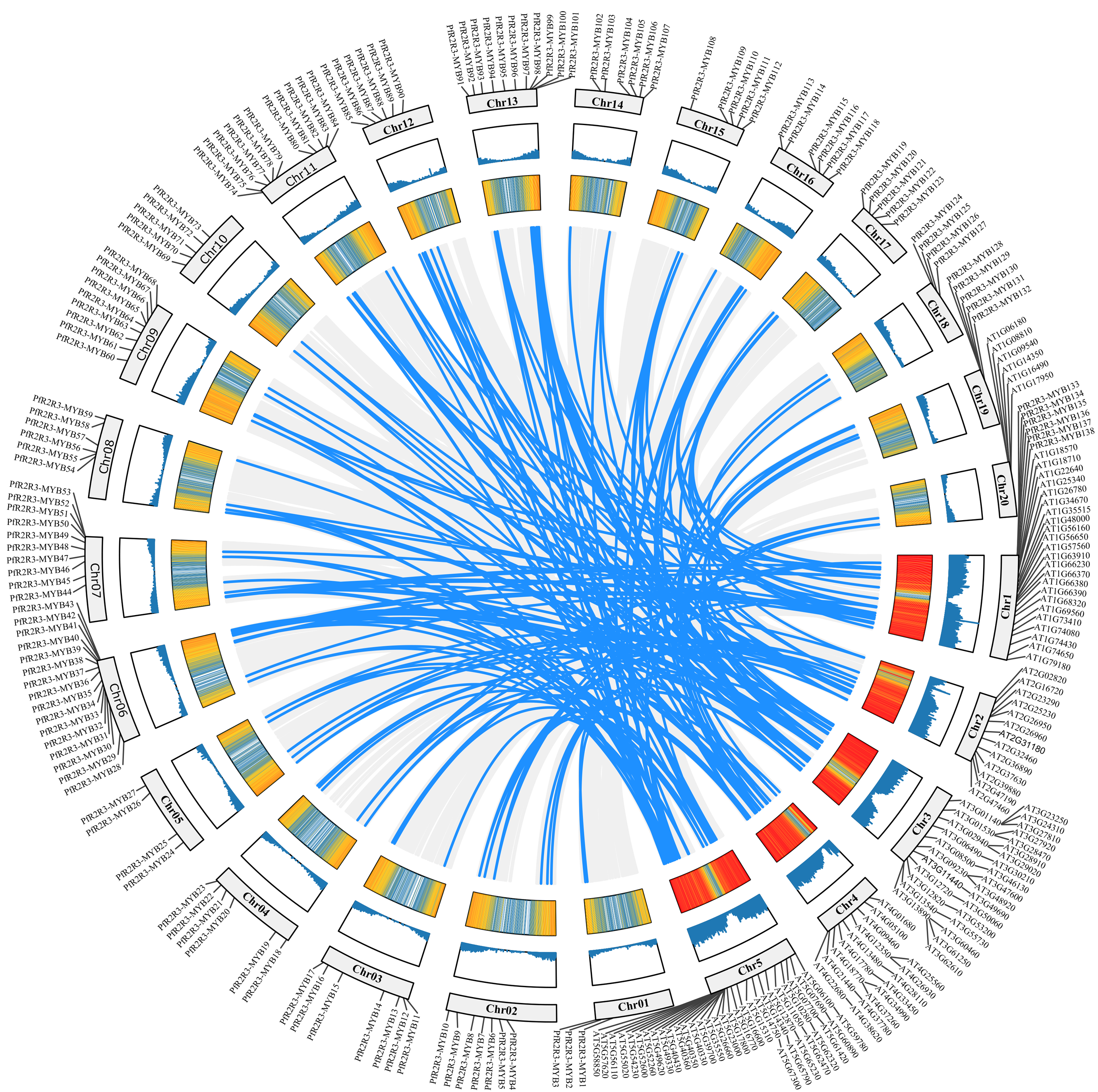

Supplement: Supplementary file 1 [file genes-14-00007-s001.zip › Supplementary Materials Figure S1.tif]

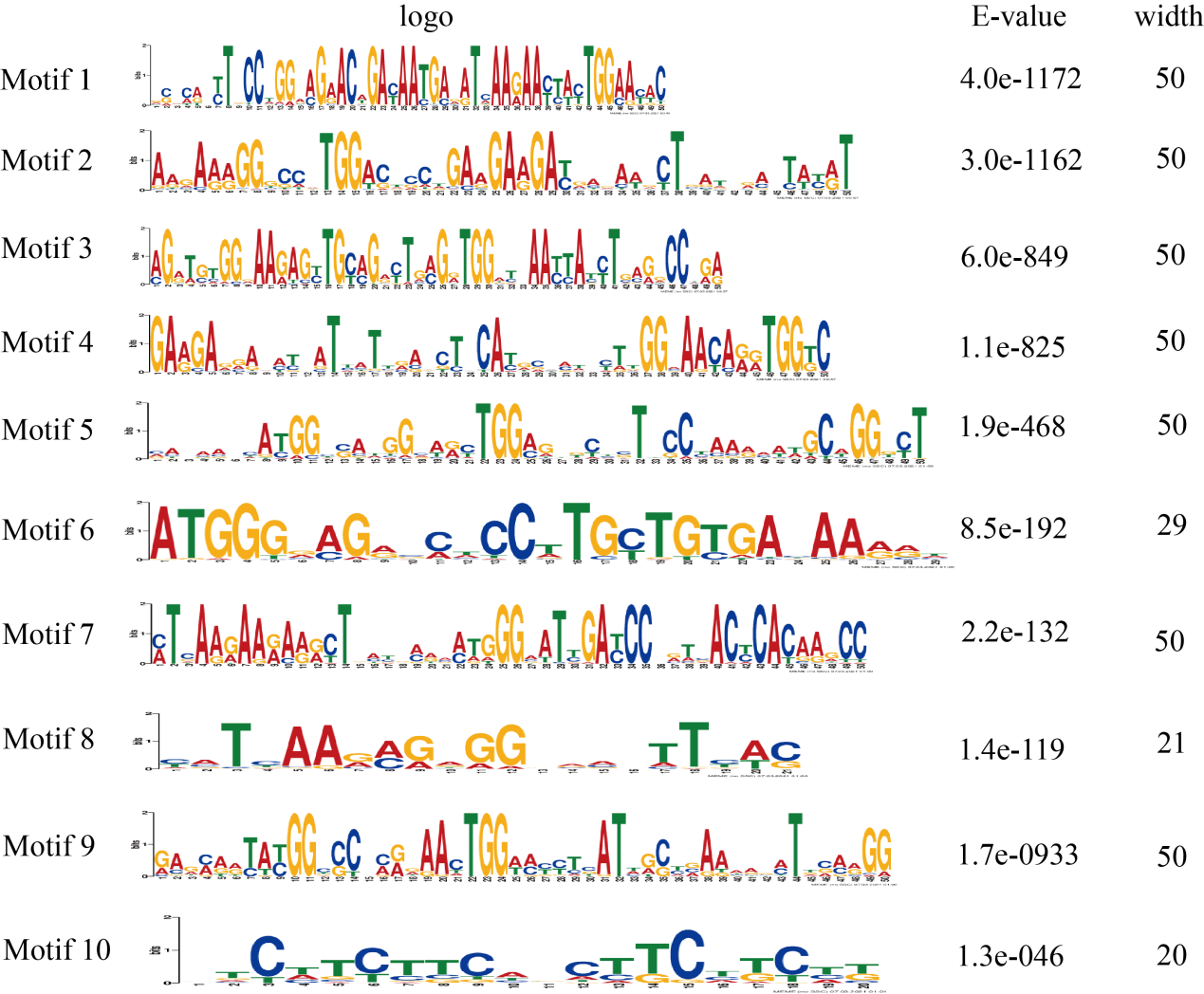

Supplement: Supplementary file 1 [file genes-14-00007-s001.zip › Supplementary Materials Figure S2.tif]

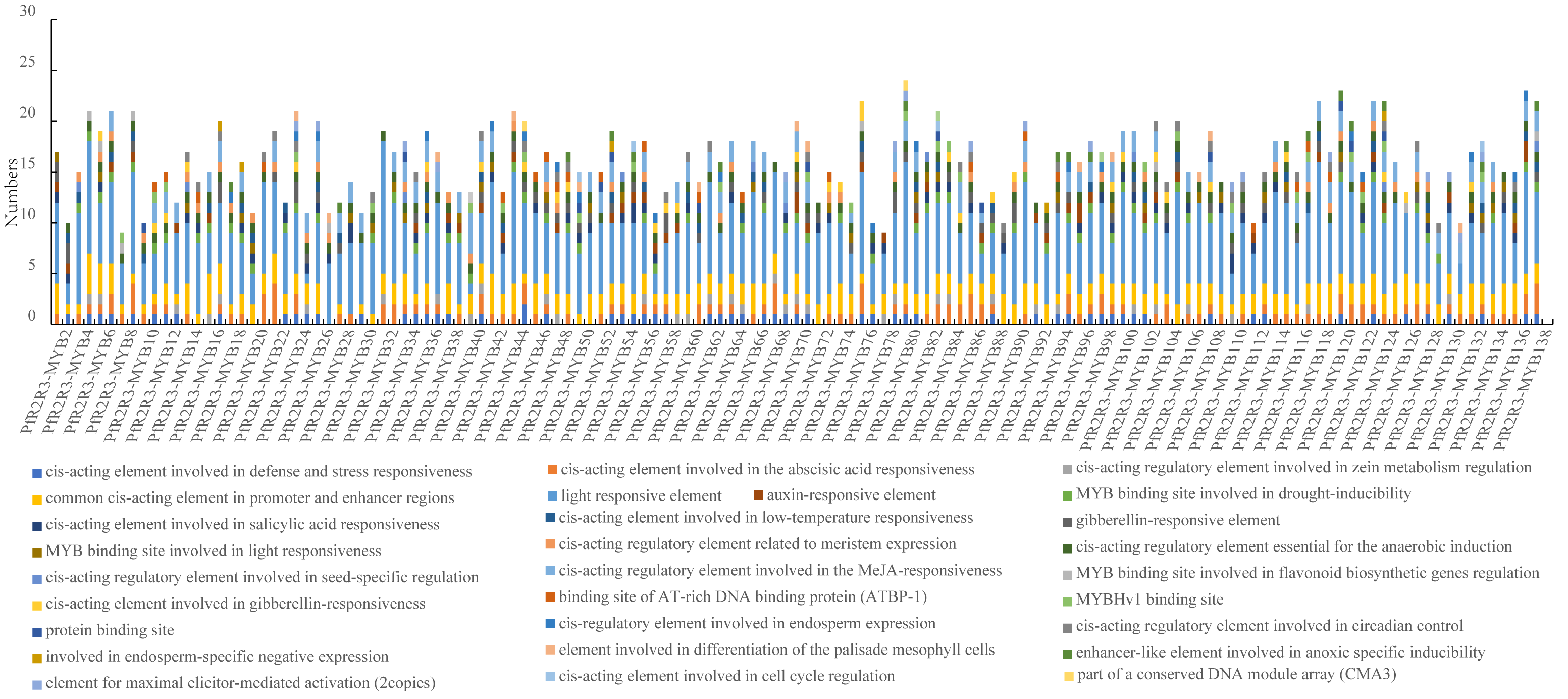

Supplement: Supplementary file 1 [file genes-14-00007-s001.zip › Supplementary Materials Figure S3.tif]
